# NPs/NPRs Signaling Pathways May Be Involved in Depression-Induced Loss of Gastric ICC by Decreasing the Production of mSCF

**DOI:** 10.1371/journal.pone.0149031

**Published:** 2016-02-10

**Authors:** Xue-Lian Lin, Xu-Dong Tang, Zheng-Xu Cai, Feng-Yun Wang, Ping Li, Hua Sui, Hui-Shu Guo

**Affiliations:** 1 Central Laboratory, The First Affiliated Hospital of Dalian Medical University, Dalian 116011, Liaoning Province, China; 2 Department of Gastroenterology, Xiyuan Hospital, China Academy of Chinese Medical Sciences, Beijing, 100091, China; 3 Department of Neurology, The First Affiliated Hospital of Dalian Medical University, Dalian 116011, Liaoning Province, China; 4 Institute of Basic Research of Integrative Medicine, Dalian Medical University, Dalian, 116044, Liaoning Province, China; University of Texas Medical Branch, UNITED STATES

## Abstract

It is well known that natriuretic peptides (NPs) are involved in the regulation of gastrointestinal motility. Interstitial cells of Cajal (ICC) are the pacemaker cells of gastrointestinal motility and gastrointestinal dyskinesia is one of the important digestive tract symptoms of depression. However, it is unclear whether they are involved in depression-induced loss of ICC. The aim of the present study was to investigate the relationship between the natriuretic peptide signaling pathway and depression-induced loss of gastric ICC in depressed rats. These results showed that the expression of c-kit and stem cell factor (SCF) in smooth muscle layers of stomach were down-regulated in depressed rats at the mRNA and protein levels. The expression of natriuretic peptide receptor (NPR)-A, B and C were up-regulated in the stomach of depressed rats at the mRNA and protein levels. NPR-A, B and C can significantly decrease the expression of SCF to treat cultured gastric smooth muscle cells (GSMCs) obtained from normal rats with different concentrations of C-type natriuretic peptide (CNP). Pretreatment of cultured GSMCs with 8-Brom-cGMP (8-Br-cGMP, a membrane permeable cGMP analog), cANF (a specific NPR-C agonist) and CNP (10^−6^ mol/L) demonstrated that 8-Br-cGMP had a similar effect as CNP, but treatment with cANF did not. The results of the methyl thiazolyl tetrazolium bromide (MTT) assay indicated that high concentrations of cANF (10^−6^ mol/L) restrained the proliferation of cultured GSMCs. Taken together, these results indicate that the up-regulation of the NPs/NPR-C and NPs/NPR-A, B/cGMP signaling pathways may be involved in depression-induced loss of gastric ICC.

## Introduction

Depression is a common mental disease. Depression patients demonstrate the following symptoms: low spirits, loss of interest, guilt or inferiority, sleep anxiety or loss of appetite, insomnia or drowsiness, fatigue or energy loss, and poor concentration or difficulty in the decision making [1]. It has been reported that gastrointestinal tract (GIT) involvement is an important predictor of depressive symptoms, and patients with a depressed mood had worse scores on self-reported GI symptoms[2]. GI dysmotility may develop as a result of an accumulation of continuous or repeated stress in some individuals[3]. Several studies have indicated that GI motility disorders, such as irritable bowel syndrome (IBS) and functional dyspepsia (FD), are associated with a high comorbidity of psychiatric disorders, particularly depression and anxiety disorders.[4–6]

Normal gastric emptying requires the coordinated function of the upper stomach fundus, lower body, antrum, and pylorus.[7] The gastrointestinal motility function and its regulation are accomplished via the collaboration and communication of extrinsic innervation to the stomach[7], enteric nerves, central nervous system[4], smooth muscle cells, interstitial cells of Cajal (ICCs)[8] and immune cells[7]. ICC was first described by the Spanish neuroanatomist Santiago Ramón y Cajal[9]. Some studies have reported that the number of ICCs and their function are damaged and difficult to reverse in various gastrointestinal motility disorders.[10–12] ICCs as pacemaker cells are distributed throughout the gastrointestinal tract[13] and have an important effect on the regulation of smooth muscle activity by producing and propagating slow waves[14]. It is well known that ICCs express the gene product of c-kit, a receptor tyrosine kinase, while SCF, the ligand for c-kit, is produced by smooth muscle cells[15]. Previous studies have shown that the development and maintenance of ICCs are dependent on SCF, particularly the membrane-bound stem cell factor (mSCF) via kit.[10,16,17] Thus, the Kit/SCF signaling pathway is essential for the proliferation of ICCs.[15,18] Furthermore, c-kit is considered to be a marker to identify ICCs, as a previous study demonstrated that ICCs express c-kit and that all c-kit cells expressed by GIT are ICCs.[19]

In 1981, de Bold *et al* isolated atrial natriuretic peptide (ANP) from the atrium [20]. Later, B-type natriuretic peptide (BNP), and C-type natriuretic peptide (CNP), dendroaspis natriuretic peptide (DNP), micrurus natriuretic peptide (MNP), as well as ventricular natriuretic peptide (VNP) were successively found. NPs are distributed over the entire body in addition to the heart and produce a variety of biological effects, such as natriuretic-diuretic[20], modulation of water and salt homeostasis, vasorelaxation[21], and decreased blood pressure[22], by binding to relevant transmembrane receptors. The following three subtypes of natriuretic peptide receptors have been identified: natriuretic peptide receptor-A (NPR-A), natriuretic peptide receptor-B (NPR-B) and natriuretic peptide receptor-C (NPR-C)[23]. NPR-A and NPR-B consist of extracellular ligand-binding domains, intercellular transmembrane domains, protein kinase homology domains, and the guanylyl cyclase domain,[24–26], which can facilitate the formation of cGMP from GTP[27]. Both ANP and BNP are preferentially coupled to NPR-A, and CNP has a much higher affinity for NPR-B[27,28]. These three natriuretic peptides indiscriminately bind to NPR-C, which is widely distributed in all kinds of tissues and does not produce cGMP[29] because it does not contain intercellular domains[26,30]. NPR-C, in addition to its clearance function[21], may mediate the cell function immediately via diversified intracellular signal transduction pathways. Recent reports have indicated that NPR-C is combined to the phospholipase C, adenylyl cyclase[31,32] and MAP kinase signaling pathways[33]. To date, many reports have suggested that NPs regulate DNA synthesis as well as cell proliferation. NPs inhibit the proliferation of several cell types, which appear to be mediated by NPR-C, including aortic smooth muscle cells[34] as well as endothelial and vascular smooth muscle cells[12,35]. Some studies have shown that NPRs are distributed in the rat gastric smooth muscle layer and that CNP relaxes the circular and longitudinal smooth muscles in the stomach of humans, rats, and guinea pigs[20,25]. Depression can appear as gastrointestinal disorders[3], while GD is associated with the depletion of ICC[10–12] and NPs/NPRs signaling pathways are participated in the regulation of the gastric motility[20,25]. Nevertheless, it is still unclear whether NPs play an effect on the depletion of ICC in depressed rats. The aim of this work was to study the effect of the NPs/NPRs signaling pathways on the survival and proliferation of ICCs and smooth muscle cells in the stomach of depressed rats.

## Materials and Methods

### Reagents and antibodies

HANK’S balanced salt mixture, 4× loading buffer, pre-stained molecular weight markers, phosphate buffer solution (PBS), Tris buffered saline, Tween-20 were purchased from Beijing Solarbio Science & Technology Co., Ltd. Antibiotic/antimycotic, Trypsin 0.25% (1×) solution and DMEM/F-12 were purchased from GE Healthcare Life Sciences HyClone Laboratories, Beijing, China. ATP and Methyl thiazolyl tetrazolium (MTT) solution were purchased from Amresco, USA. Rabbit anti-SCF antibody, rabbit anti-c-kit and rabbit anti-β-actin were purchased from Beijing Biosynthesis Biotechnology Co., Ltd. Rabbit polyclonal to NPR-A, rabbit polyclonal to NPR-B, and rabbit monoclonal to NPR-C were purchased from Abcam (Shanghai) trading Co., Ltd (Abcam Shanghai Office). Horseradish peroxidase-conjugated goat anti-rabbit secondary antibody was purchased from Beijing ZSGB Biotech Co., Ltd. RNAiso Plus, PrimeScript^™^ RT reagent kit and SYBR^®^ Premix Ex Taq^™^ II were purchased from Takara Biotech, Inc. Other reagents were purchased from local distributors such as Dalian Chen Yu Biotechnology Co. Ltd. China.

### Animals and the depression rat model

Male Sprague-Dawley (SD) rats (License No.: SCXK (LIAO) 2008–0002, and certificate No. 0003496) of clean grade (about 180g) were provided by the Laboratory Animal Center of Dalian Medical University. They were randomly divided into two groups: the normal comparison group (N) and depression model group (M).

The animals were free to get food and water and were maintained under standard housing conditions (temperature 25±2°C, relative moisture 69±2%) with a 12-hour light and dark cycle. Rats were maintained under previously described conditions for 1 week before they were subjected to the experiments. On the basis of the previous research methods, group M were singly housed and received one of the chronic mild unpredictable irritations daily: restraint, swim induced fatigue, footshock, fasting and concussion[1]. This study was performed with the approval of the Animal Ethics Committee of Dalian Medical University and all the experiments were performed according to the Guideline for the Care and Use of Laboratory Animals published by the Science and Technology Commission of the People’s Republic of China (STCC Publication No. 2, revised 1988).

### Depression model identification

Conditions such as spirits, vitality and the color of ear mucosa were observed every day. The measure of weight, sucrose preference test and open-field test were performed before modeling and at the end of the experiment. At the end of the experiment, the gastric residual rate was tested.

### Gastric smooth muscle cell culture

Entire stomachs were rapidly excised from normal SD rats and placed in ice-cold HANK’S solution. These stomachs were dissected along the greater curvature, while the submucosal and mucosal layers were removed carefully in HANK’S solution containing 10% antibiotic/antimycotic. The smooth muscle layers of stomach were rinsed with cold and germ-free PBS three times for at least 3 min each. Then, the tissues were minced into small pieces. GSMCs were spread by enzymatic digestion in a 1-ml solution, including 1.3 mg of collagenase II, 2 mg of bovine serum albumin, 2 mg of trypsin inhibitor and 0.27 mg of ATP at 37°C, stirring the digestion for 60 minutes, and the cells were collected every 30 min. Next, culture medium containing fetal bovine serum (FBS) was added to terminate the digestion. Cells were dispersed from the tissues by gentle trituration using a narrow-bore blunt glass pipette and cultured in DMEM/F-12 with 15% FBS and a 1% antibiotic/antimycotic solution at 37°C in a humidified incubator with 5% CO_2_. The culture medium was replaced every 3 days, and the cells were passaged every 5–7 days. Immunohistochemistry showed that the cell cultures were α-actin-positive, such that the cells were confirmed to be smooth muscle cells. GSMCs between the fifth to sixth passages were used for the experiments. Prior to the experiments, the medium was replaced with serum-free medium for an additional 24 h when the GSMCs grew to confluence. The GSMCs were washed 3 times with germ-free PBS and then incubated with DMEM/F-12-containing 0.5% FBS and different concentrations of drugs. Control GSMCs were cultured with DMEM/F-12-containing 0.5% FBS in the absence of any drugs.

### Western blotting analysis

Protein samples were extracted from frozen gastric tissue and were cultured with GSMCs according to the manufacturer’s instructions in RIPA lysis buffer (P0013B, Beyotime). Total protein samples were blended with 4× loading buffer in 100°C water for 5 min prior to the protein assay. Protein and pre-stained molecular weight markers were separated using 6% and 10% SDS-polyacrylamide gel electrophoresis and transferred onto polyvinylidene difluoride (PVDE) membranes (Millipore, Bedford, MA). The membrane was soaked in 5% skimmed dried milk in Tris buffered saline-Tween 20 (TBST) for one hour at 37°C to block the membrane and then incubated with primary antibodies, including a rabbit polyclonal to NPR-A (ab70848), rabbit polyclonal to NPR-B (ab14357), rabbit monoclonal to NPR-C (ab177954), rabbit anti-SCF antibody (bs-0545R), rabbit anti-c-kit (bs-0672R) and rabbit anti-β-actin (bs-0061R) in TBST at 4°C overnight. A horseradish peroxidase-conjugated goat anti-rabbit secondary antibody (ZB-2301) was used at a dilution of 1:10000 in TBST at room temperature for 60 min. Blotting membranes were immersed in chemiluminescent detection reagent (R-03031-C50, R-03025-C50; advansta) for approximately 3 min. After ECL development, each western blot image was quantitatively analyzed using Quantity One software (FluorChem FC3) and normalized against β-actin.

### Fluorescence quantitative real-time polymerase chain reaction (RT-PCR) analysis of NPR-A, NPR-B, NPR-C, SCF and c-kit gene expression

Total RNA was extracted from the gastric smooth muscle layer in the antrum and body according to the manufacturer’s instructions for RNAiso Plus (9109,TAKARA BIO INC). After testing the concentration of RNA, the sample concentration was adjusted to 1 μg/μl according to the instruction manual for PCR amplification. Single stranded cDNA was synthesized in a 20-μl reaction mixture, containing 1 μl of total RNA, 4 μl of 5× PrimeScript RT Master Mix and 15 μl of RNase-free dH_2_O. Reverse transcription was performed according to the instruction of PrimeScript^™^ RT Master Mix (RR036A; TAKARA BIO INC.), which was incubated for 15 min at 37°C, followed by incubation for 5 s at 85°C. cDNA samples were used to analyze specific cDNA of NPR-A, NPR-B, NPR-C, SCF and c-kit. Each multiplex PCR contained 1 μl of cDNA sample, 3 μl of RNase-free dH_2_O, 5 μl of SYBR Premix Ex TaqII (Tli RNaseH Plus) (2×), 0.2 μl of ROX Reference Dye (50×), 0.4 μl of sense primer, and 0.4 μl of anti-sense primer. The following conditions were applyed for each PCR amplification: Stage 1: Reps 1, 95°C for 30 s; Stage 2: Reps 40, 95°C for 5 s; 60°C for 30 s, which was selected according to the instruction manual of SYBR^®^ Premix Ex TaqTM II (RR820A; TAKARA BIO INC.) using the StepOnePlus^™^ System (Applied Bio-systems). Specific primers for rat β-actin, NPR-A, NPR-B, NPR-C, SCF and c-kit were synthesized by the Bioneer Biological Company (Bioneer, Inc., South Korea, Table 1). Gene expression of NPR-A, NPR-B, NPR-C, SCF and c-kit were normalized against β-actin expression. The relative increase/decrease of mRNA of target gene in the experimental group was calculated using the normal group as the calibrator using the Ct values and ⊿⊿ Ct value of the amplification system.

**Table 1:** Primer sequences used for real time PCR.

Gene	^a^Sense	^b^Antisense
β-actin	5’-TATCGGCAATGAGCGGTTCC-3’	5’-AGCACTGTGTTGGCATAGAGG-3’
NPR-A	5’-CCTTTCAGGCTGCCAAAAT-3’	5’-ATCCTCCACGGTGAAGTTGA-3’
NPR-B	5’-TTTACCTTGATGTCTTTGGG-3’	5’-CCTGATACTCGGGATTCG-3’
NPR-C	5’-GGCGGCTCAAAAGATCGAG-3’	5’-CCATTGCCCTCCACGCTAC-3’
SCF	5’-GGGAGTGAGCCCTTATGCCA-3’	5’-TGTCCAATCCACCCGAACT-3’
c-kit	5’-TGAAGATGCTCAAACCAAGTGC-3’	5’-CGCTCCAAGGAGGTTGACGATA-3’

^a^Sense: Forward primer sequence.

^b^Antisense: Reverse primer sequence.

### MTT assay

Cell proliferation was measured using the MTT assay kit according to the manufacturer’s instruction. Briefly, GSMCs were trypsinized into a cell solution and grown in a 96-well plate at 1.5×10^5^ cells/ml (2×10^4^ cells per well) and DMEM/F-12-containing 15% FBS for 24 h. Next, the medium was replaced with DMEM/F-12, including 0.5% FBS. After a 24-h culture, the cells were exposed to 15% FBS / DMEM/F-12 with different concentrations of cANF (10–8, 10–7, 10–6 mol/L; Bachem), respectively, and cultured for 24 h at 37°C in a humidified atmosphere, containing 5% CO_2_. Each concentration was tested in no fewer than 15 wells. Next, 20 μL of MTT solution (final concentration 0.5 mg/mL) was added to each well and the plate was further incubated for 4 h at 37°C to deoxidize MTT under light-blocking conditions. After removal of the medium and MTT dye solution, 150 μL of dimethyl sulfoxide (DMSO) was added into each well and shaken for 10 min at room temperature to dissolve all of the crystals. The absorbance (A) values at 490 nm were measured using a Microplate spectrophotometer.

### Statistical analysis

The data of all of the analytes are presented as the mean±SE. Differences between the groups were analyzed using the Independent-Samples T-test and one-way ANOVA. A *p*-value less than 0.05 was considered statistically significant. The database was established using the SPSS 13.0 software package from SPSS, Inc.

## Results

### Establishment and identification of the animal model of depression

The performance, weight and open-field test of all rats were used to identify if the depression model was successful. The performances of normal rats were the following: good spirits; activity was agile; thick and shiny hair; ear mucosa was pale pink; and eating, drinking and stool were normal. The performances of the depressed rats were as follows: demeanor was exhausted; hair was dull and brown; dark eyes had secretions; arched and huddled together; and stool was loose. Loss of weight and a reduction in the number of crossed-grids, standing and grooming were also observed.

**Fig 1:**
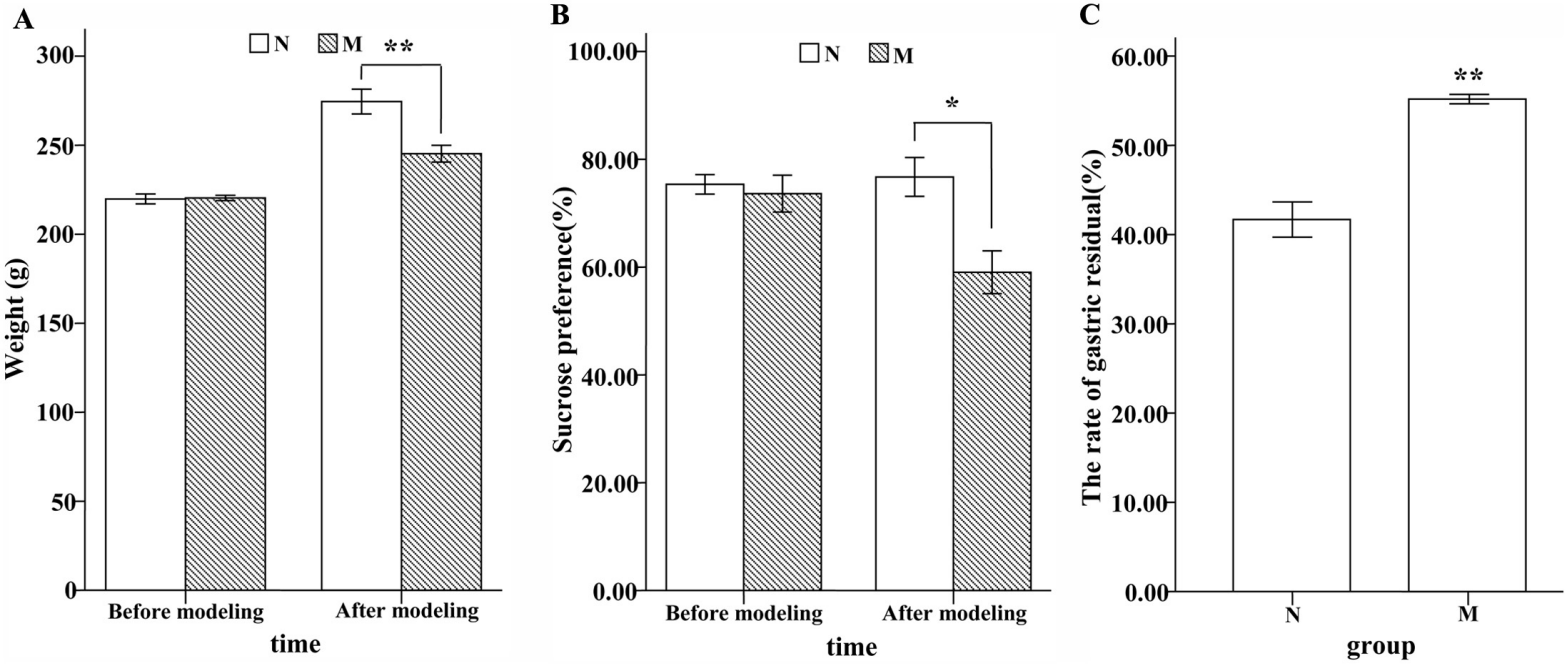
Rats show depressive-like symptoms at the end of the experiment. (A) Body weight. (B) Percentage of sucrose solution from the total liquid in sucrose preference test. The results of the percentage were 59.05±3.97 in group M and 76.73±3.60 in group N. (C) The rate of gastric residual. The results were 55.18±0.52 in M and 41.68±1.97 in N.

The weight of the rats: Before modeling, there was no significant difference between group N and group M. On the 27^th^ day, the average weight of rats was 274.50±6.96 in N and 245.22±4.75 in M, respectively. The difference between M and N was significant (**P<0.01, Independent-Samples T-test; Fig 1A). Rats in group M exhibited significantly lower sucrose preference compared with rats in group N (*P<0.05, Independent-Samples T-test, Fig 1B) at the end of the experiment. The rate of gastric residual was higher in group M, which indicated that gastric emptying was decreased, compared with group N (**P<0.01, Independent-Samples T-test; Fig 1C).

**Fig 2:**
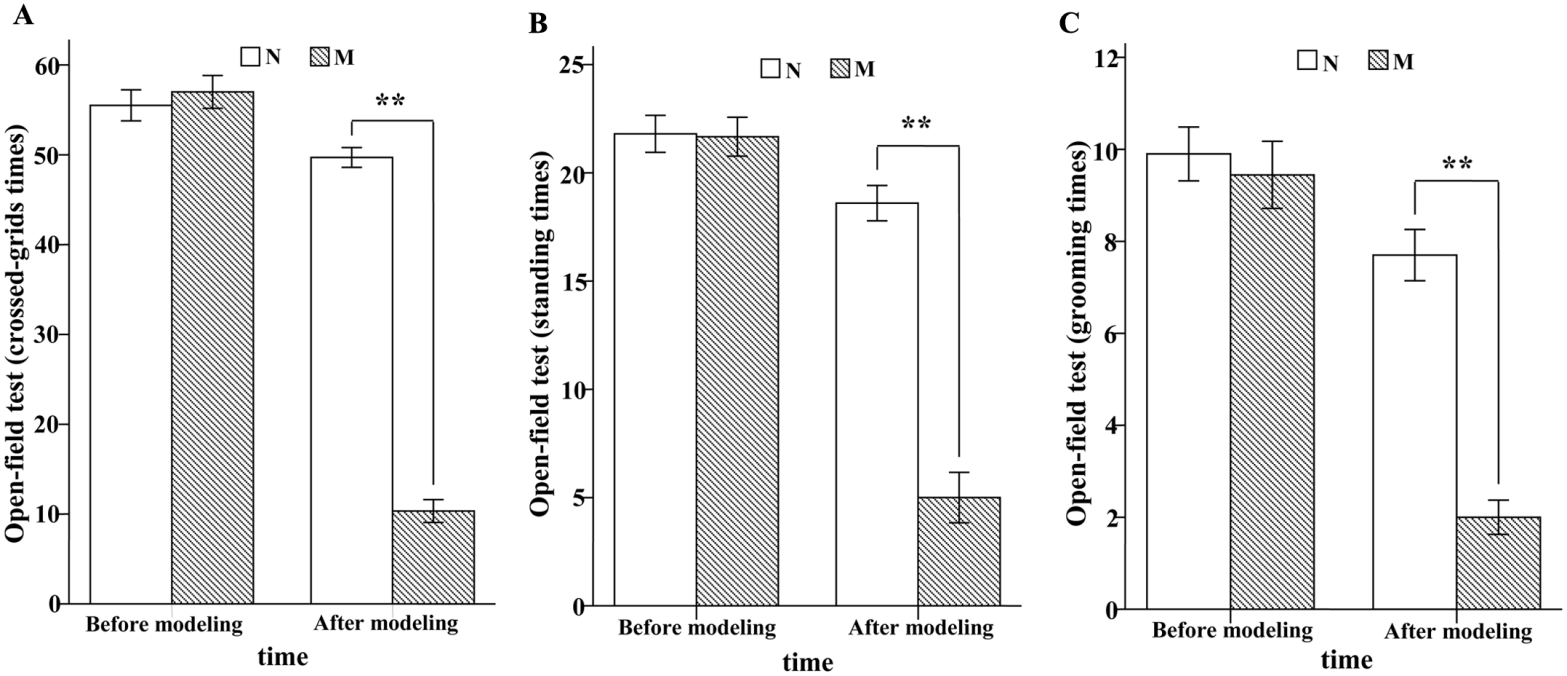
The result of open-field test. (A) Crossed-grids times. At the end of the experiment, the average crossed-grids times were 10.33±1.27 in M and 49.70±1.12 in N. (B) Standing times. At the end of the experiment, the average standing times were 5.00±1.17 in M and 18.60±0.82 in N. (C) Grooming times. At the end of the experiment, the average grooming times were 2.00±0.37 in M and 7.70±0.56 in N.

Open-field test: The number of crossed-grids, standing and grooming were compared. At the beginning, there was no significant difference between M and N. At the end of the experiment, the difference between M and N was significant (**P<0.01, Independent-Samples T-test; Fig 2), which indicated that the locomotor activity of rats in group M was decreased significantly compared with group N.

### Expression of c-Kit and SCF in gastric smooth muscle layers

**Fig 3:**
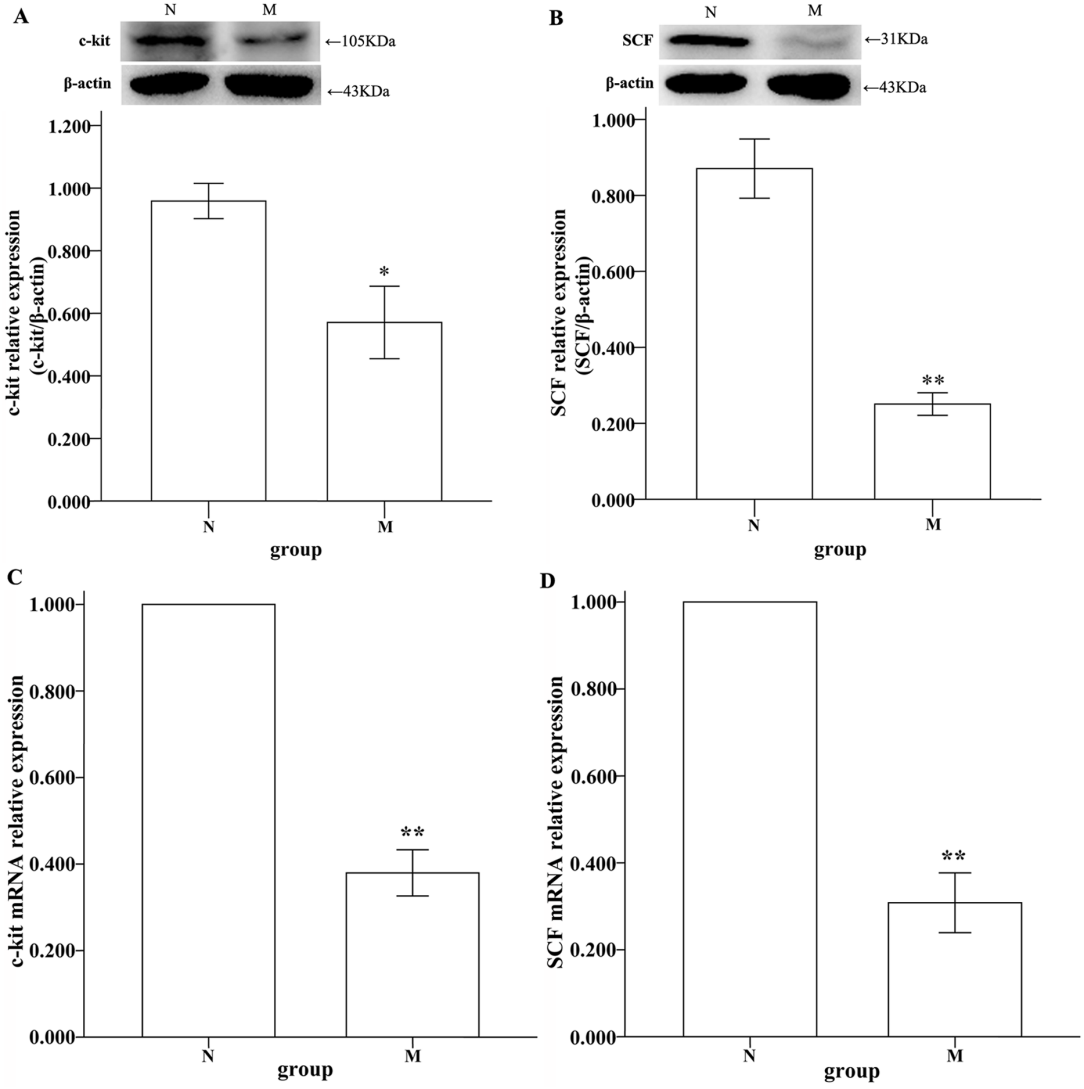
Western blotting and real-time PCR analyses of SCF and c-kit in the stomachs of rats. (A) The average ratio of c-kit/β-actin was 0.959±0.056 in N and 0.571±0.116 in M. (B) The average ratio of SCF/β-actin was 0.871±0.078 in N and 0.251±0.030 in M. The differences between M and N were significant (compared with normal, *P<0.05, Independent-Samples T-test, A and **P<0.01, Independent-Samples T-test, B). (C-D) The result of c-kit is 1.000±0.000 in N and 0.380±0.053 in M. The result of SCF is 1.000±0.000 in N and 0.308±0.069 in M. The differences between N and M were significant (**P<0.01, Independent-Samples T-test).

It is well established that ICC can trigger the spontaneous rhythmic contractions of smooth muscle in stomach. Thus, loss of ICCs and disruption to their networks are likely to play an important role in various gastrointestinal motility disorders in patients and animal models[11]. As a receptor tyrosine kinase, c-kit was used as a marker of ICC[19]. In this study, the protein and gene expression levels of c-Kit and SCF in the smooth muscle layers of gastric antrum and corpus were examined using western blotting and RT-PCR analyses. Both c-Kit and SCF protein and the gene expression levels were significantly down-regulated in depressed rats (compared with normal rats, *P<0.05, Independent-Samples T-test; Fig 3A; **P<0.01, Independent-Samples T-test; Fig 3B, 3C and 3D).

### NPR expression in gastric smooth muscle layers

**Fig 4:**
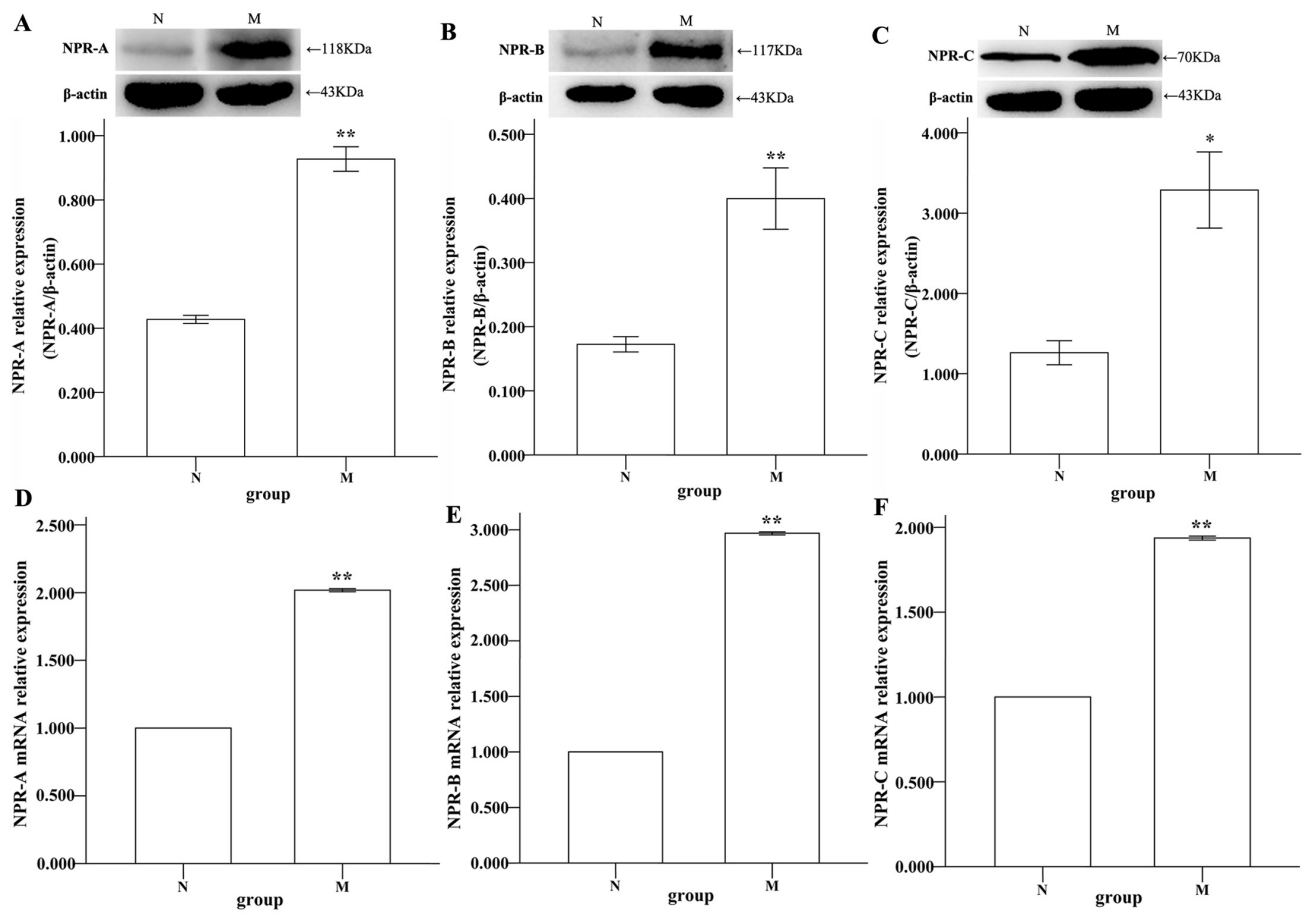
Western blotting and real-time PCR analyses of NPR-A, B and C in the stomachs of rats. (A-C) The ratio of NPR-A/β-actin was 0.427±0.013 in N and 0.927±0.038 in M. The ratio of NPR-B/β-actin was 0.172±0.012 in N and 0.400±0.048 in M. The ratio of NPR-C/β-actin was 1.261±0.151 in N and 3.287±0.474 in M. The differences between M and N were significant (compared with normal, **P<0.01, Independent-Samples T-test, A-B; *P<0.05, Independent-Samples T-test, C). (D-F) The result of NPR-A is 1.000±0.000 in N and 2.019±0.005 in M. The result of NPR-B is 1.000±0.000 in N and 2.969±0.012 in M. The result of NPR-C is 1.000±0.000 in N and 1.936±0.012 in M. The differences between N and M were significant (**P<0.01, Independent-Samples T-test).

The NPR transcript levels were quantified employing the method of real-time PCR and normalized against the β-actin transcript levels in each sample. The values of ΔΔ Ct were expressed as the ratio of the normal group expression level. These results showed that M and N both expressed NPRs, but gene expression was significantly increased in the depressed rat (**P <0.01, Independent-Samples T-test; Fig 4D–4F).

Western blotting analyses were performed to detect the protein expression of NPRs in the smooth muscle layer of gastric antrum and corpus of depressed rats. The molecular weights were the following: NPR-A 118 kDa, NPR-B 117 kDa and NPR-C 70 kDa. These results showed that M and N both expressed the NPR protein, but the expression levels were up-regulated in depressed rats (**P<0.01, Independent-Samples T-test, Fig 4A and 4B; *P<0.05, Independent-Samples T-test, Fig 4C).

### Impact of the NPR agonist on the expression of SCF in cultured GSMCs

**Fig 5:**
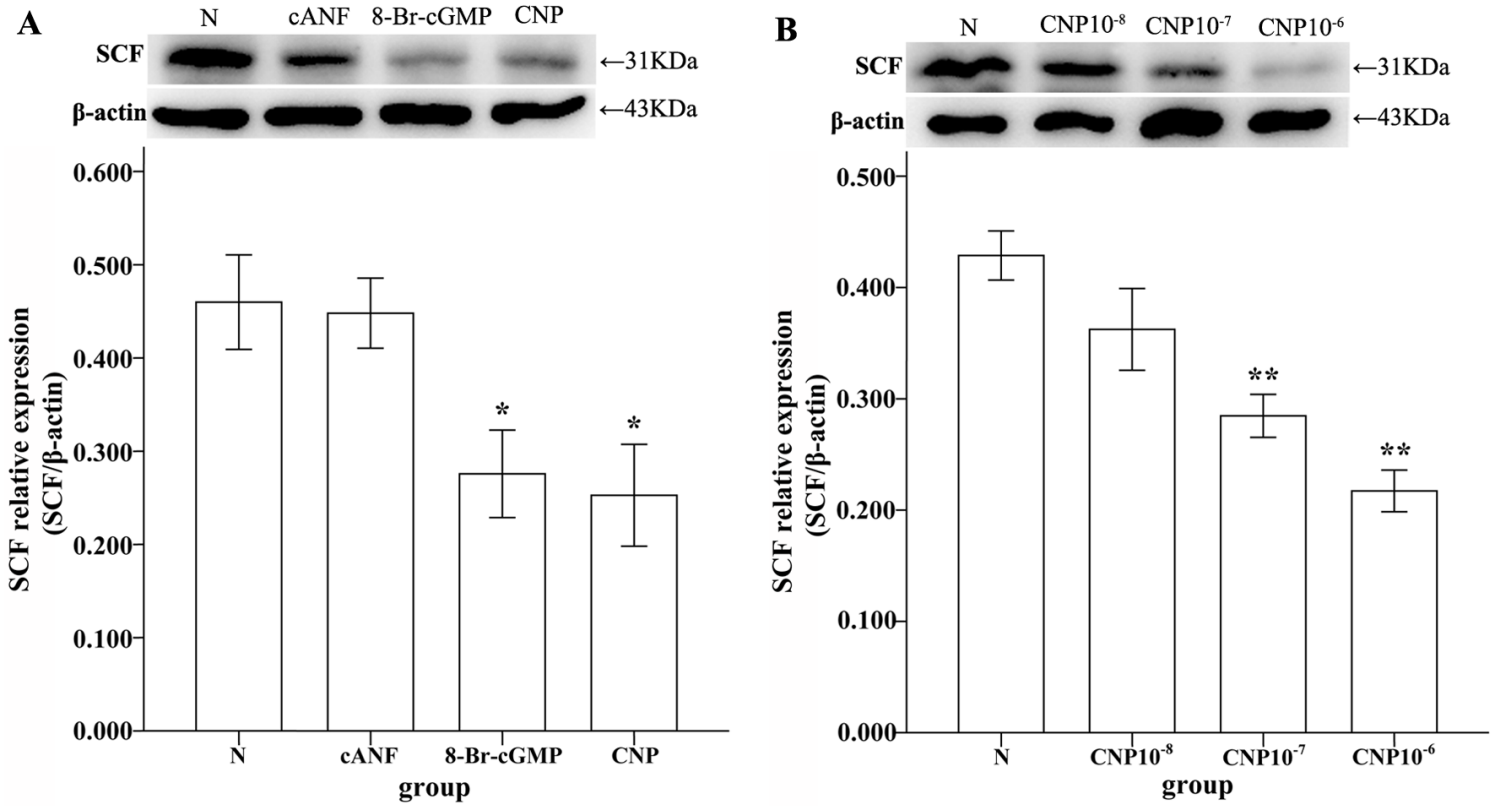
Western blotting analyses of SCF expression in cultured GSMCs with different pretreatment conditions. (A) The ratio of SCF/β-actin was 0.460±0.051 in N, 0.448±0.038 in cANF(10^−6^ mol/L) group, 0.276±0.047 in 8-Br-cGMP (10^−6^ mol/L) group and 0.253±0.055 CNP (10^−6^ mol/L) group. The expression levels were significantly different in the 8-Br-cGMP (10^−6^ mol/L) group and CNP (10^−6^ mol/L) group (*P<0.05, one-way ANOVA), but not in the cANF(10^−6^ mol/L) group (P>0.05, one-way ANOVA) compared with N. (B) The ratio of SCF/β-actin was 0.429±0.022 in N, 0.362±0.037 in CNP (10^−8^ mol/L) group, 0.282±0.020 in CNP (10^−7^ mol/L) group, and 0.217±0.019 CNP (10^−6^ mol/L) group. These results showed that high concentrations of CNP (10^−7^ mol/L and 10^−6^ mol/L) could significantly decrease the expression of SCF (**P<0.01, one-way ANOVA).

Because CNP can bind to three NPRs (particularly, NPR-B and NPR-C), we treated cultured GSMCs obtained from normal rats and observed the effect of CNP on SCF expression to investigate the relationship between SCF production and up-regulated NPRs. The CNP-treated group was cultured with medium containing different concentrations of CNP. Control GSMCs were cultured using the same culture medium in the absence of any drugs for 48 h. Next, the culture medium was removed and the GSMCs were lysed with RIPA lysis buffer. Western blotting analysis showed that the expression level of SCF was significantly decreased by CNP (10^−7^, 10^−6^ mol/L) (**P<0.01, one-way ANOVA; Fig 5B). The GSMCs were treated with cANF (10^−6^ mol/L), 8-BrcGMP (10^−6^ mol/L) and CNP (10^−6^ mol/L) to investigate which NP signaling pathway had a major effect on this result. These results indicated that cANF could not change the expression level of SCF (P>0.05, one-way ANOVA), while 8-Br-cGMP and CNP demonstrated similar alterations on the expression level of SCF (*P<0.05, one-way ANOVA; Fig 5A).

### Effect of cANF on the proliferation of cultured GSMCs

**Fig 6:**
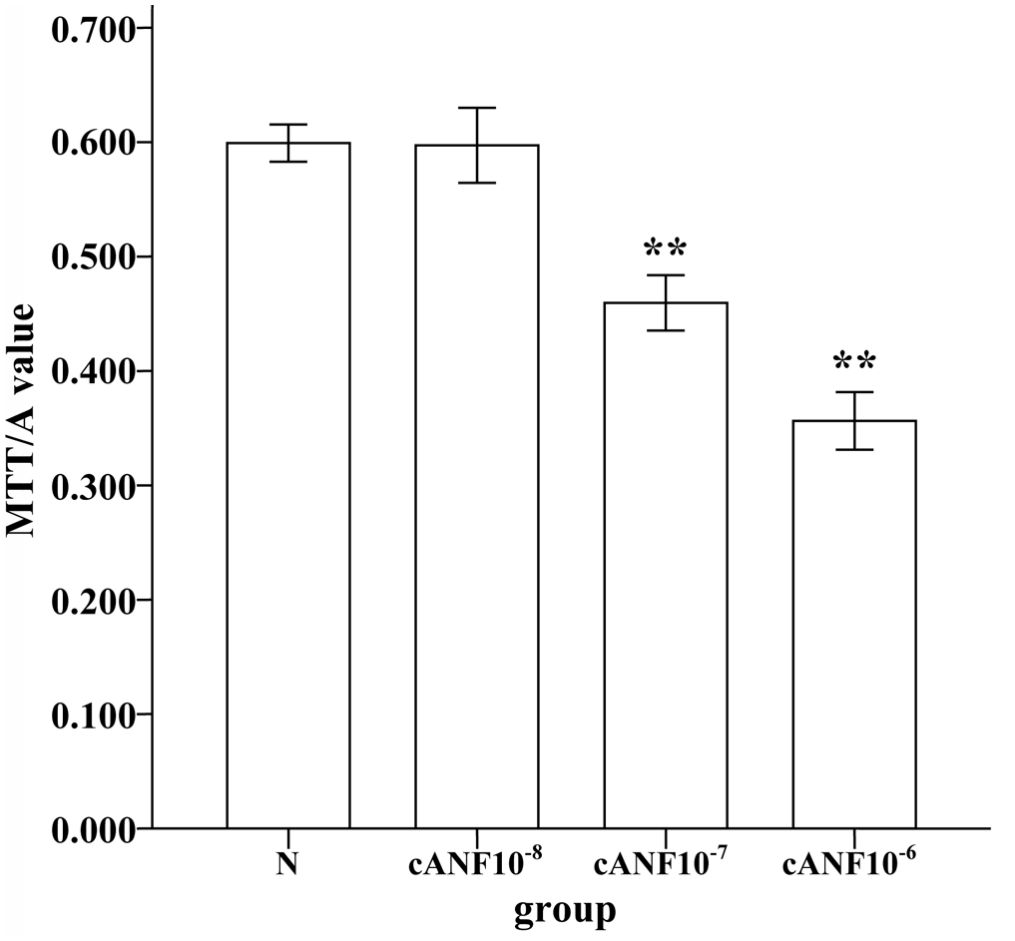
Effects of cANF on A value of MTT of rat GSMCs. cANF The average value was 0.599±0.016 in control group, 0.597±0.033 in 10^−8^ mol/L group, 0.459±0.024 in 10^−7^ mol/L group and 0.356±0.025 in 10^−6^ mol/L group.

NPRs are significantly up-regulated in depression; thus, we aimed to investigate whether NPs affect the proliferation of GSMCs. The results obtained using the MTT assay showed that the A490 value in cANF-treated GSMCs was reduced compared with the control group at concentrations of 10^−6^ mol/L and 10^−7^ mol/L (**P<0.01, one-way ANOVA; Fig 6), and there was no significant difference at 10^−8^ mol/L (P>0.05, one-way ANOVA; Fig 6). These results indicated that higher concentration of cANF (10^−6^ mol/L) inhibited the proliferation of GSMCs via the NPR-C signaling pathway.

## Discussion

Depressive disorder is a common mental disorder and has been identified as a leading cause of burden in the Global Burden of Disease (GBD) 2010.[36] Gastrointestinal dysfunction is a common complication of depression, and approximately half of patients with functional gastrointestinal disorders (FGID) also demonstrate symptoms of depression and/or anxiety disorders.[37] ICCs, which are located between the gut nerve fibers and smooth muscle cells, serve as electrical pacemakers and generate spontaneous electrical slow waves in the gastrointestinal tract.[10,38] Furthermore, ICCs contribute to several other important functions in the GIT, such as the transduction of motor neural inputs from the enteric nervous system and mechanosensation to stretch the GI muscles, among other functions.[39] Thus, loss of ICCs or injury to ICC networks may be strongly associated with various chronic GI motility disorders. A number of studies have shown that the development, survival and maintenance of ICCs is dependent on SCF signaling via c-kit.[16,17] First, we aimed to demonstrate whether the number of ICCs was altered via changes in the c-kit and SCF expression levels in depressed rats. These results showed that the expression of c-kit was consistently decreased at the level of the gene and protein, which indicated that the number of ICCs was remarkably decreased in the stomachs of depressed rats. Moreover, the SCF expression levels were also decreased in these rats. Thus, we can infer that loss of gastric ICCs and down-regulation of the SCF/c-kit signaling pathway occurs during depression.

CNP was first extracted from porcine brain in the 1990s[12] and is considered to be a peptide that co-exists in the central nervous system and gastrointestinal tract, playing a bioactive role in the brain-gut axis[40]. It has been reported that CNP relaxes gastric longitudinal and circular smooth muscles in the stomachs of humans, rats and guinea-pigs by activating its cognate receptor, NPR-B.[20,25] CNP signals its activity via three types of natriuretic peptide receptors (particularly, NPR-B and NPR-C). Our previous study has shown that the CNP/NPR-B signal pathway is up-regulated in the rectum of depressed rats[1]. However, it is still unclear whether the NP/NPR signaling pathway changes or whether there is a relationship between depletion of ICC and changes in the natriuretic peptide signaling pathway in the stomachs of depressed rats. Our results showed that all three types of receptors are significantly increased in the stomachs of depressed rats at both mRNA and protein levels.

Different concentrations of CNP were used to treat GSMCs cultured from normal rats, and the expression of SCF was detected using western blotting analyses to investigate the relationship between the up-regulated of NP signaling pathway and the production of SCF. Western blotting analysis revealed that CNP decreased the expression of SCF in cultured GSMCs. It is difficult to find inhibitors of NPRs, and thus, 8-Br-cGMP (10^−6^ mol/L), cANF (10^−6^ mol/L) and CNP (10^−6^ mol/L) were added to GSMCs to further elucidate the mechanism. Our results demonstrated that 8-Br-cGMP had a similar effect with CNP, but not cANF. These findings showed that up-regulation of NPRs in the stomachs of depressed rats may decrease the production of SCF via the NP/NPR-A,B/cGMP signaling pathway and further indirectly affect the survival and function of ICC.

It has been reported that NPs regulate DNA synthesis and the proliferation of several cell types, such as endothelial and vascular smooth muscle proliferation, and this function may be mediated by NPR-C[35]. On this basis, we further investigated whether the natriuretic peptide signaling pathway could affect the proliferation of GSMCs after it was up-regulated. These results showed that high concentrations of cANF, a specific NPR-C agonist, inhibited the proliferation of GSMCs. Because SCF is produced by GSMCs[15], inhibition of GSMC proliferation could affect the production of SCF and further alter SCF/c-kit signaling pathway.

In conclusion, our findings showed that the SCF/c-kit signaling pathway was down-regulated and that the natriuretic peptide signaling pathways were up-regulated in the stomachs of depressed rats. Moreover, the CNP/NPR-A and B/cGMP signaling pathways decreased the SCF expression levels, while the cANF/NPR-C signal pathway decreased the production of SCF by indirectly inhibiting the proliferation of gastric smooth muscle cells. Thus, we concluded that the up-regulation of the NPs/NPR signaling pathways may be involved in depression and induce the loss of gastric ICC via an indirect decrease in the production of SCF.

## Supporting Information

S1 Table (A) The weight of rats (mean±SE). (B) Sucrose preference test(%). (C) The rate of gastric residual(%).
(DOCX)

S2 Table (A) Open-field test(crossed-grids times). (B) Open-field test (standing times). (C) Open-field test (grooming times).
(DOCX)

S3 Table Effects of cANF on *A* value of MTT of rat GSMCs (mean±SE). (DOCX)
