# Supplementary material for: NPs/NPRs Signaling Pathways May Be Involved in Depression-Induced Loss of Gastric ICC by Decreasing the Production of mSCF
Source: PLoS One. 2016 Feb 10;11(2):e0149031. doi: 10.1371/journal.pone.0149031 (PMC4749124; doi:10.1371/journal.pone.0149031)
Supplement: S1 Table — (A) The weight of rats (mean±SE). (B) Sucrose preference test(%). (C) The rate of gastric residual(%). (DOCX) [file pone.0149031.s001.docx]

**Table. A. The weight of rats (mean±SE)**

|  | Before modeling | After modeling |
| --- | --- | --- |
| N | 219.80±2.77 | 274.50±6.98 |
| M | 220.38±1.70 | 245.22±4.75 |

**Table. B. Sucrose preference test (%)**

|  | Before modeling | After modeling |
| --- | --- | --- |
| N | 75.36±1.82 | 76.73±3.60 |
| M | 73.63±3.43 | 59.05±3.97 |

**Table. C. The rate of gastric residual (%)**

| N | 41.68±1.97 |
| --- | --- |
| M | 55.18±0.52 |
