# Supplementary material for: NPs/NPRs Signaling Pathways May Be Involved in Depression-Induced Loss of Gastric ICC by Decreasing the Production of mSCF
Source: PLoS One. 2016 Feb 10;11(2):e0149031. doi: 10.1371/journal.pone.0149031 (PMC4749124; doi:10.1371/journal.pone.0149031)
Supplement: S2 Table — (A) Open-field test(crossed-grids times). (B) Open-field test (standing times). (C) Open-field test (grooming times). (DOCX) [file pone.0149031.s002.docx]

**Table. A. Open-field test (crossed-grids times)**

|  | Before modeling | After modeling |
| --- | --- | --- |
| N | 55.50±1.73 | 49.70±1.11 |
| M | 57.00±1.83 | 10.33±1.27 |

**Table. B. Open-field test (standing times)**

|  | Before modeling | After modeling |
| --- | --- | --- |
| N | 21.80±0.85 | 18.60±0.82 |
| M | 21.67±0.90 | 5.00±1.17 |

**Table. C. Open-field test (grooming times)**

|  | Before modeling | After modeling |
| --- | --- | --- |
| N | 9.90±0.59 | 7.70±0.56 |
| M | 9.44±0.73 | 2.00±0.37 |
