# Supplementary material for: NPs/NPRs Signaling Pathways May Be Involved in Depression-Induced Loss of Gastric ICC by Decreasing the Production of mSCF
Source: PLoS One. 2016 Feb 10;11(2):e0149031. doi: 10.1371/journal.pone.0149031 (PMC4749124; doi:10.1371/journal.pone.0149031)
Supplement: S3 Table — (DOCX) [file pone.0149031.s003.docx]

**Table. Effects of cANF on *A* value of MTT of rat GSMCs (mean±SE)**

| Group | Control | 10^-8^mol/L cANF | 10^-7^mol/L cANF | 10^-6^mol/L cANF |
| --- | --- | --- | --- | --- |
| MTT/ *A* value (n=15) | 0.599±0.016 | 0.597±0.033 | 0.459±0.024 | 0.356±0.025 |
